# Machine-Learning-Based Color Sensing Using Wearable SENSIPATCH Spectrometer Module: An Experimental Study

**DOI:** 10.3390/s26092576

**Published:** 2026-04-22

**Authors:** Hamza Mustafa, Federico Fina, Mario Molinara, Luigi Ferrigno, Andrea Ria, Paolo Bruschi, Simone Contardi, Fabio Leccese, Hafiz Tayyab Mustafa

**Affiliations:** 1 Department of Electrical and Information Engineering, University of Cassino and Southern Lazio, 03043 Cassino, Italy; m.molinara@unicas.it (M.M.); ferrigno@unicas.it (L.F.); 2 Sensichips s.r.l., Via delle Valli 46, 04011 Aprilia, Italy; simone.contardi@phd.unipi.it; 3 Department of Science, Roma Tre University, 00146 Rome, Italy; federico.fina@uniroma3.it (F.F.); fabio.leccese@uniroma3.it (F.L.); 4 Department of Information Engineering, University of Pisa, 56100 Pisa, Italy; andrea.ria@unipi.it (A.R.); paolo.bruschi@unipi.it (P.B.); 5 School of Computer Science and Technology, Zhejiang Normal University, Jinhua 321004, China; mustafa.tayyab@zjnu.edu.cn; 6 Zhejiang Institute of Photoelectronics, Jinhua 321004, China

**Keywords:** machine learning, spectroscopy, sensors, LED, wearable, colors, PANTONE, color sensing, classification

## Abstract

Accurate color classification plays a critical role across diverse fields, from textile manufacturing and environmental monitoring to biomedical diagnostics. This study introduces a machine-learning-driven approach to spectral color sensing using SENSIPATCH, a compact, wearable sensor system; while SENSIPATCH integrates multiple sensing modalities, including bioimpedance, electrochemical, thermal, humidity, and vibrational sensors, this work specifically utilizes its spectrometer module, which comprises multi-wavelength LEDs and photodiodes. Targeting the classification of 100 standardized PANTONE colors, the proposed framework is evaluated under controlled lighting conditions to ensure repeatable spectral acquisition. The experimental design includes both firm and loose contact scenarios to emulate variability in wearable placement. A structured data-preprocessing pipeline involving baseline correction, bootstrapping, and Z-score normalization was employed to enhance signal quality and improve model generalization. Five machine learning models were evaluated: Random Forest, SVM, MLP, CNN, and LSTM. The MLP demonstrated the strongest classification performance. Notably, the MLP achieved consistent accuracy across both contact conditions, indicating robustness against sensor placement variations. These results highlight the feasibility of compact LED-based wearable spectroscopy for reliable color classification under controlled measurement conditions, providing a baseline for future extensions to more diverse lighting conditions.

## 1. Introduction

**Figure 1:**
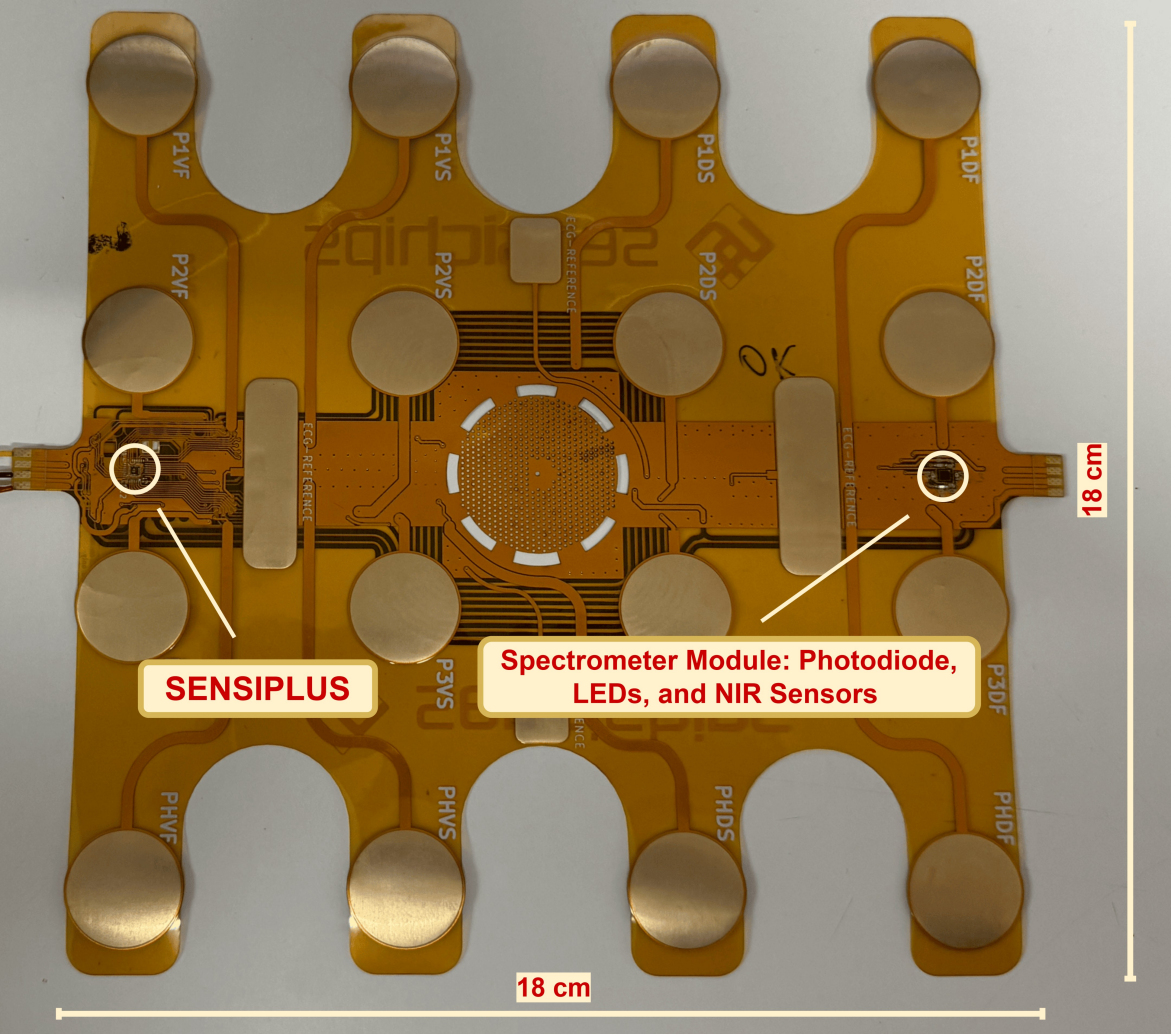
SENSIPATCH wearable device.

**Figure 2:**
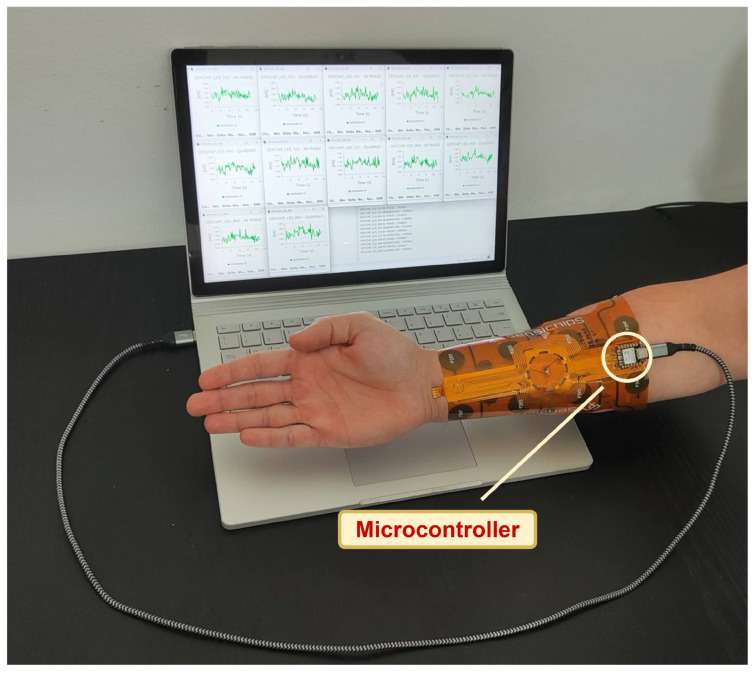
Illustration of the SENSIPATCH wearable patch placed on the arm.

Color classification is a fundamental challenge in numerous domains, including art [1], product design [2], agriculture [3], environmental monitoring [4], and biomedical diagnostics [5]. Across these industries, precise and consistent color identification underpins quality control, regulatory compliance, and functional effectiveness. For instance, in the textile and design sectors, accurate reproduction of standard color palettes is vital for customer satisfaction [6,7], while in biomedicine [8] and agrifood industries [9,10,11], color signatures often serve as proxies for health or ripeness assessment. Traditional techniques for color classification, such as visual inspection and handheld colorimeters, suffer from subjectivity, limited spectral resolution, and poor repeatability across varying lighting conditions. Spectrometry-based approaches [12,13], though significantly more accurate, are constrained by high costs, bulkiness, and the need for controlled laboratory environments. Consequently, there is a growing demand for miniaturized, portable, and cost-effective solutions capable of delivering high-fidelity spectral measurements in real-time, in real contexts. In parallel, recent advances in compact multichannel optical filtering structures have demonstrated highly selective wavelength separation across the visible to near-infrared range, further highlighting the potential of miniaturized optical platforms for sensing applications [14]. Emerging innovations in discrete LED-based spectroscopy offer an appealing alternative. Unlike traditional spectrometers, LED-based systems utilize fixed-wavelength light sources to achieve spectral measurements with reduced complexity, making them ideal for wearable and embedded systems. Among such systems, the SENSIPATCH [15,16] as shown in Figure 1, developed by Sensichips [17], represents a cutting-edge solution for wearable spectral sensing. This 18 cm × 18 cm patch integrates a scattering-based spectrometer and the SENSIPLUS [18] single-chip versatile sensor interface, enabling real-time spectral analysis across visible and near-infrared ranges. As shown in Figure 2, the SENSIPATCH is worn directly on the arm, offering an ergonomic and portable form factor suitable for continuous use.

**Figure 3:**
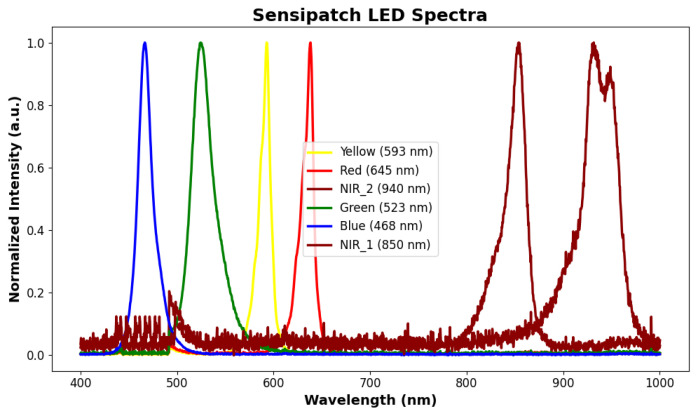
Normalized emission spectra of the six LEDs used in the SENSIPATCH spectrometer module.

The system spectrometer module operates using four discrete LEDs (Blue: 468 nm, Green: 523 nm, Yellow: 593 nm, Red: 645 nm) and two NIR photodiodes (850 nm and 950 nm), supported by a central photodiode for light intensity capture. To compensate for the limited number of LEDs while still covering a broad portion of the visible and near-infrared spectrum (Figure 3), the acquired current signals were analyzed using machine learning models. This approach allows the classification framework to exploit the combined information from all spectral channels for discrimination among the different PANTONE classes considered in this study.

This configuration provides broad spectral coverage while maintaining low power consumption and form-factor efficiency. Additionally, the device incorporates a 16-electrode bioimpedance matrix supporting spectroscopy from 10 Hz to 1.5 MHz, enabling the monitoring of body composition, hydration levels, respiration, and cardiovascular activity. Environmental parameters are captured using Al_2_O_3_-based gas sensors for sweat and vapor detection, as well as integrated relative humidity and temperature sensors. Electrochemical capabilities are provided via an embedded potentiostat/galvanostat for voltammetry, while an audio frequency response sensor (7 Hz–1 kHz) supports mechanical and vibrational signal capture.

**Figure 4:**
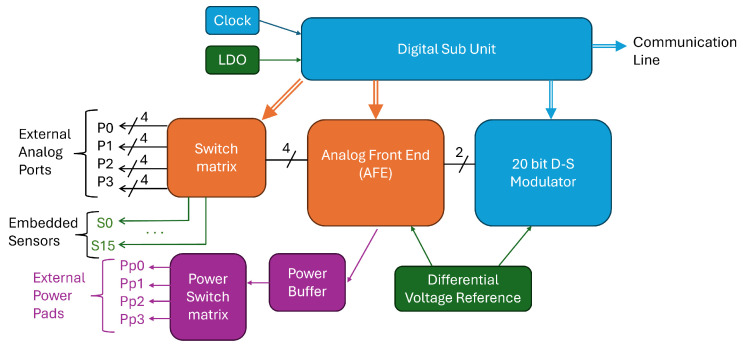
Simplified block diagram of the SENSIPLUS versatile sensor interface.

The core of the proposed system is represented by SENSIPLUS. It is a fully programmable, single-chip integrated sensor interface that operates at low voltage and is fabricated using the UMC 0.18 μm CMOS process. Detailed information about its features can be found in [19], while here we recall the main ones. A simplified block diagram of the SENSIPLUS interface is shown in Figure 4. The system includes a programmable analog front end (AFE) for both stimulating the sensor and reading its response. A Digital Sub Unit oversees system operation and facilitates communication with an external host via the Communication Line, using standard protocols such as SPI, I2C, or I3C, or alternatively through a proprietary single-wire protocol called SENSIBUS. The SENSIPLUS is capable of acquiring signals from either external sensors connected to its analog terminals (P0–P3) or from its integrated sensors (S0–S15). Additional External Power Pads (Pp0–Pp3) can be used to stimulate devices that require currents in the order of tens of mA. As in the presented works, LEDs are connected to these pads. The acquisition of LEDs responses is performed relying on a single photodiode, whose response is converted in the digital domain by means of the embedded 20-bit analog-to-digital converter. Building upon prior applications of the SENSIPLUS microsensor in ECG monitoring [20], optical spectroscopy [21], and water pollutant detection [22,23], this work extends its utility to the domain of color science.

In this study, we specifically utilize the SENSIPATCH’s spectrometer module to collect color spectral data from standardized PANTONE [24] color card samples. The aim is to exploit the platform’s multispectral sensing capabilities for accurate color classification, with data subsequently analyzed using machine learning techniques to develop a robust, wearable-compatible color recognition approach. The PANTONE color cards were used as physical reference samples, placed directly on the sensor surface to ensure consistent and standardized data collection across a broad spectrum of colors. As a globally recognized and standardized color system used across textiles, printing, plastics, and digital media, PANTONE [25,26] offers a controlled and reproducible palette, making it ideal for benchmarking color classification systems. Its discrete, industry-standard definitions ensure that spectral variations can be accurately linked to specific hues, enabling a rigorous and repeatable evaluation of the sensor’s performance. Moreover, the wide diversity of shades in the PANTONE library allows us to test the classifier’s sensitivity to subtle color differences, including pastel tones, deep pigments, and near-neutral grays. The primary objectives of this study are:

- To evaluate the accuracy and robustness of the SENSIPATCH spectrometer module for classifying 100 PANTONE color samples under controlled lighting conditions, with the aim of enabling future extensions to unconstrained or variable lighting scenarios.
- To develop a robust end-to-end machine learning solution capable of operating on wearable-acquired spectral data.
- To investigate the impact of physical contact variation (firm vs. loose contact) on classification accuracy, simulating real-world wearable deployment scenarios.
- To explore the feasibility of using compact, LED-based spectroscopy for scalable deployment in applications beyond color classification, such as biomedical diagnostics, quality control, and environmental monitoring.

Building on earlier preliminary investigations [16] on PANTONE color classification with the SENSIPATCH system, this work considers a larger standardized dataset, includes two contact conditions, adopts a more detailed preprocessing procedure, and provides a broader comparative evaluation of machine learning models.

The rest of the paper is structured as follows: Section 2 reviews recent literature on LED-based spectral sensing and color classification. Section 3 describes the experimental protocol and data processing methods. Results and model evaluations are presented in Section 4, followed by conclusions and future directions in Section 5.

## 2. Related Work

Recent years have witnessed significant developments in the integration of LED-based spectroscopic sensing and machine learning for applications ranging from environmental monitoring and biomedical diagnostics to food quality assessment and industrial automation.

### 2.1. LED-Based Optical Sensing and Spectrophotometry

Low-cost LED-based optical systems have gained traction as alternatives to bulky spectrometers. Marsan et al. [27,28] demonstrated reliable spectrophotometric measurements using geometrical optics and real-time processing. Rosa et al. [29] advanced this by developing an optofluidic micro device that integrates RGB LEDs with multispectral detection for automated spectrophotometric analysis. Similarly, Rocha et al. [30] proposed a visible-light-based LED system to measure multiphase liquid levels, using ML to identify fluid interfaces in industrial contexts. Fragner et al. [31] proposed an indoor positioning system using LEDs as both emitters and sensors, coupled with machine learning for enhanced localization, demonstrating the versatility of LED-based optical systems beyond illumination.

### 2.2. Wearable and Non-Invasive Sensing Systems

Wearable and portable LED-based systems have found promising applications in biomedical domains. Hina et al. [32] developed a single-LED photoplethysmography system for non-invasive glucose monitoring, while Manurung et al. [33] integrated NIR sensors with IoT and ML for smart, portable blood glucose tracking. Crafa et al. [34] evaluated the trade-offs between digital and analog embedded neural networks for color recognition in wearable systems, focusing on power and latency optimization. Tobar et al. [35] introduced Skinly, a handheld IoT spectrophotometric device for analyzing skin properties using ML. Similarly, Hsu et al. [36] designed a mobile solution to measure human skin spectra via low-cost sensors and deep neural networks. Khan et al. [37] developed an IoT-enabled diabetes management system using non-invasive glucose monitoring via near-infrared and bio-impedance spectroscopy, combined with ML algorithms.

### 2.3. Food Quality, Botanical, and Environmental Applications

Machine learning combined with NIR and LED sensors has proven effective in food classification and botanical studies. Zeb et al. [38] used NIR LED wavelengths (770–960 nm) for fruit type determination via ML. Sulistyo et al. [39] developed a portable LED spectrometer for detecting coconut sugar adulteration. Hanopol et al. [40] applied LED-ML frameworks to detect rice tungro disease, confirming the relevance of light-absorption profiles in agricultural health. Ueno et al. [41] proposed a supervised learning calibration method for color-sensitive sensors in botanical settings. Greco et al. [42] used terahertz imaging for non-invasive measurement of plant water content, showcasing high-resolution optical sensing in agriculture. Tamayo-Monsalve et al. [43] utilized convolutional neural networks and transfer learning to classify coffee fruit maturity stages using multispectral images, demonstrating the effectiveness of deep learning in agrifood spectral analysis. Greco M. et al. [44] made THz measurements to distinguish healthy and rotten hazelnut samples.

### 2.4. Colorimetric and Chemical Sensing

Numerous efforts have focused on ML-based LED systems for chemical and color detection. Goyal et al. [45] created a colorimetric sensor for chlorine and pH detection using LED-LDR configurations and ML regressors. Batinic et al. [46] proposed spectrum estimation improvements via neural networks applied to reflected colorimetric signals. Lee et al. [47] introduced an ultra-low-power micro-LED-based gas sensor integrated with deep learning for real-time VOC detection in an e-nose configuration.

### 2.5. Vision-Based and Hyperspectral Imaging Techniques

Advanced imaging techniques have also contributed to color and spectral classification. Zhu et al. [48] employed hyperspectral imaging and ML for automatic facial skin color classification, accounting for lighting variability. Li et al. [49] developed a random forest-based method to classify garment colors from online fashion images, demonstrating improved robustness over traditional methods. Garcia et al. [50] proposed an LED-based colorimetry system for identifying graffiti on urban surfaces, applying spectral analysis to public safety and forensic tasks.

### 2.6. Light Modeling and Night Vision Applications

Fan et al. [51] proposed a model for predicting spectral power distribution (SPD) of full-spectrum white LEDs using neural networks and genetic algorithms. Niu et al. [52] introduced a VIS-NIR LED-based spectral illumination framework optimized for low-light vision applications, enabling enhanced object visibility while preserving human compatibility in dark conditions. Tang et al. [53] further extended LED-based imaging with a hyperspectral solution tailored for object recognition in low-light environments.

Although several earlier studies have shown the promise of LED-based optical sensing and machine learning, their scope differs from that of our study. For example, Marsan et al. [27,28] and Rosa et al. [29] developed LED-based spectrophotometric systems, but did not examine wearable color classification on a standardized large-scale color dataset. Crafa et al. [34] explored color recognition in wearable systems, mainly from the perspective of embedded neural network implementation and efficiency trade-offs. Likewise, Batinic et al. [46], and Garcia et al. [50] addressed reflected colorimetric and LED-based color sensing in task-specific settings. In contrast, our study focuses on wearable multispectral classification of 100 standardized PANTONE classes, explicitly analyzes the effect of contact variability, and combines this with a structured preprocessing pipeline and a comparative evaluation of multiple machine learning models.

## 3. Methodology

**Figure 5:**
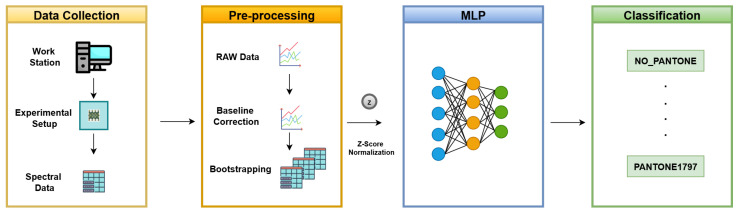
Methodology pipeline for PANTONE color sensing using data collected from the SENSIPATCH wearable system and analyzed with an MLP-based classification approach.

Our proposed methodology consists of four key phases: establishing a controlled experimental environment, collecting spectral data, preprocessing the acquired data, and applying machine learning algorithms for accurate color classification. Figure 5 presents the methodology pipeline used for this study.

### 3.1. Experimental Setup

To ensure precise and reproducible spectral data collection, we designed an experimental setup that minimizes ambient light interference and maintains consistent sensor contact. This setup consists of a custom-built black-box enclosure lined with black paper to create a controlled lighting environment, eliminating external light variations. Controlled lighting conditions are essential in spectral analysis, as variations in ambient illumination can introduce inconsistencies in sensor readings [54]. The SENSIPATCH wearable system was placed inside this enclosure to simulate real-world close-contact configurations, where the sensors interact directly with the color sample. This setup aimed to replicate practical deployment scenarios where wearable optical sensors operate in skin-contact environments, ensuring high signal fidelity and reducing external interference.

#### 3.1.1. Experimental Setup Under Varying Contact Conditions

To evaluate the impact of sensor contact pressure on spectral measurements, data collection was conducted under two different conditions:

- Firm Contact Condition (FCCW)—With Weight: A small circular weight with a hollow center (resembling a ring) with a mass of approximately 100 g, corresponding to a force of about 0.981 N and a nominal contact pressure of approximately 9.81 kPa over an effective contact area of 1.0 cm^2^, was placed on the PANTONE color card to ensure uniform and consistent pressure between the sensor and the card. The hollow center design ensures that the weight does not obstruct the light emitted by the sensors, allowing unobstructed spectral readings. This condition simulates close-contact scenarios found in wearable sensors, where firm contact enhances spectral signal stability by minimizing air gaps.
- Loose Contact Condition (LCCW)—Without Weight: The PANTONE color card was placed on the sensor without additional pressure. This scenario simulates potential variations in real-world use, where wearable sensors might experience slight detachment from the surface due to movement or external disturbances.

These two conditions were introduced to assess the impact of contact pressure on spectral measurement accuracy, as variations in sensor alignment and surface proximity can influence spectral reflectance readings.

**Figure 6:**
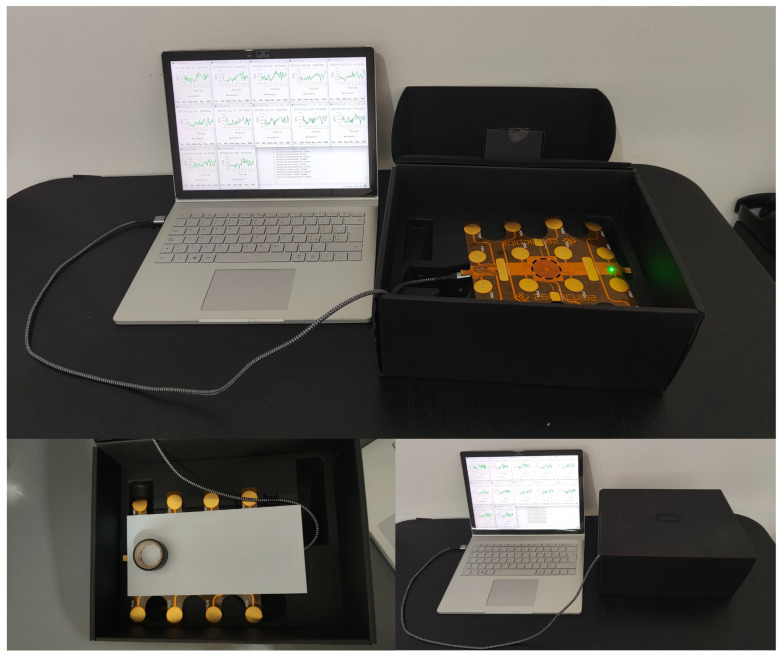
Experimental Setup used for Data Acquisition.

**Figure 7:**
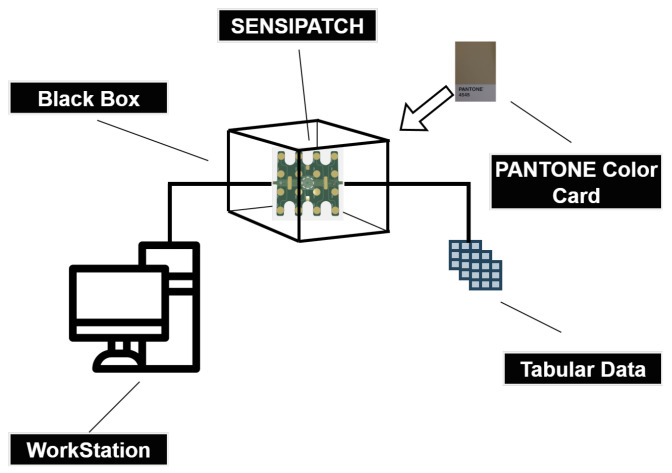
Schematic representation of the experimental setup.

Figure 6 represents the experimental setup used for this study. Figure 7 presents the schematic representation of the experimental setup used for this study.

#### 3.1.2. Measurement Procedure

A structured measurement sequence was implemented to ensure consistency in data acquisition. The following steps were performed for each experimental condition:

- Positioning the SENSIPATCH: The SENSIPATCH was placed inside the enclosure, and the lid was closed to eliminate ambient light interference.
- Baseline Measurement Collection: The sensors initially operated without any external material for the first 21 measurements, capturing baseline spectral readings under controlled conditions. These baseline measurements were subsequently used as an offset to stabilize the remaining measurements during preprocessing step.
- Placement of PANTONE Color Card: After the 21st measurement, a PANTONE color card was carefully placed on the sensor surface under the predefined contact condition (either with or without weight). The lid was closed again to maintain a controlled environment.
- Spectral Data Acquisition: Measurements resumed from the 22nd to the 121st reading to capture the spectral response of the color card under controlled conditions.
- Repetition Across Multiple Color Cards: The process was repeated for 100 different PANTONE color cards, ensuring statistical robustness in the collected dataset.

This controlled approach ensures that spectral variations can be attributed solely to the interaction between the sensor and the PANTONE color card, rather than external environmental factors. The black-box enclosure and close-contact design enhance the reproducibility of measurements, reducing errors associated with stray light or sensor misalignment.

### 3.2. Data Collection

To build a robust dataset for PANTONE color classification using the SENSIPATCH wearable system, spectral data were collected from 100 distinct PANTONE color cards and a plain white paper sample. In addition, a baseline class was recorded with the sensor exposed to ambient conditions without any object placed on it, resulting in a total of 102 classes. The complete list of PANTONE color codes included in the dataset is presented in Table 1. The data acquisition process was carefully designed to ensure measurement consistency and reliability across all samples. Each color card was subjected to five independent trials, each consisting of 121 individual sensor readings, yielding a comprehensive dataset of spectral responses for each class. To ensure accurate measurements and eliminate distance-related variability, each PANTONE card was placed in direct contact with the sensor surface, maintaining a consistent zero-gap configuration.

For each measurement sequence, the sensors were initially exposed to ambient conditions, without any PANTONE color card, in order to establish a baseline response. Following this baseline acquisition, a color card was positioned on the sensors, and data collection continued for 121 readings. This protocol was designed to capture potential sensor drift over time, thereby enhancing the accuracy and reliability of the dataset used for machine learning-based classification. In total, measurements were recorded for 100 distinct PANTONE color cards, a white paper reference, and a baseline condition representing the absence of any material on the sensors. Under two experimental conditions, with five repeated trials per card and 121 readings per trial, the final dataset comprised 123,420 samples (61,170 per condition; 102 × 5 × 121 × 2). The data acquisition rate was optimized to ensure efficiency without compromising accuracy. Each photodiode response was recorded approximately every 265 milliseconds, with all six sensors being sequentially acquired, leading to an overall sampling rate of approximately 1.6 s per complete sensor cycle.

**Figure 8:**
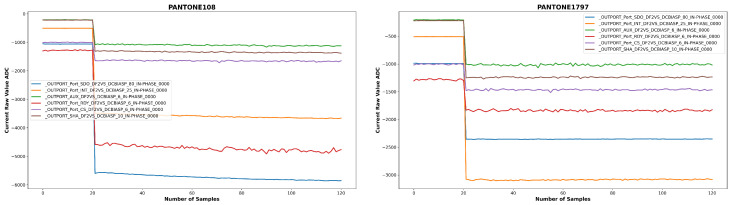
Pantone 1797 and Pantone 108 Sensipatch measurement.

The SENSIPATCH wearable system’s spectrometer operates with a lock-in amplifier. Optically, the six LEDs emit light in the VIS-NIR spectrum with a 120° emission angle. The emitted light strikes the colored surface of the PANTONE sample, which reflects it according to the spectral characteristics of the corresponding PANTONE code. The reflected light is then captured by the central photodiode, which converts it into a current signal acquired using the SLM-Studio software (version 1.2.5). The currents generated by the photodiode in response to the light reflected from the PANTONE surface under illumination by the six LEDs are the input for the machine learning models. In fact, as can be observed in Figure 8 for the PANTONE 108 (Yellow) and PANTONE 1797 (Red), the currents associated with the two NIR signals (OUTPORT_AUX and OUTPORT_SHA) vary, and so they have a diagnostic value for the purposes of PANTONE color classification by the machine learning models used in the paper. So in this case, the AUX and SHA values have small variations between the two PANTONEs on the order of hundreds of micro-Amperes.

**Figure 9:**
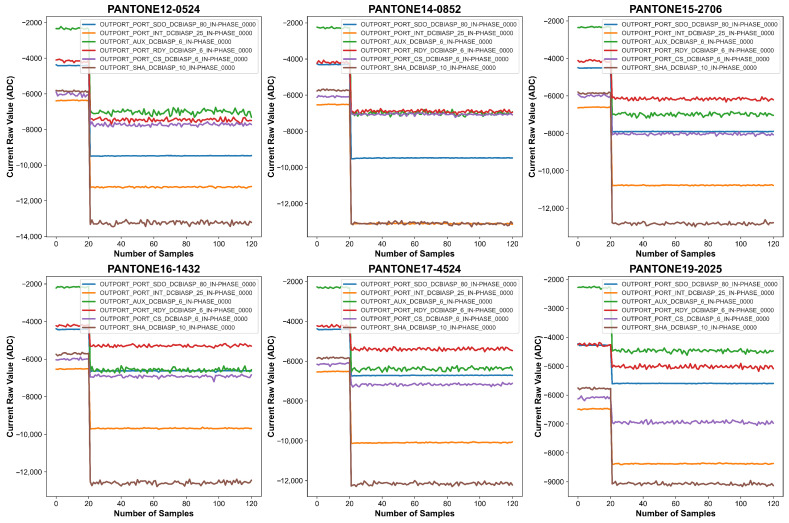
Graphical representation of the data collected for six PANTONE color cards with FCCW.

**Figure 10:**
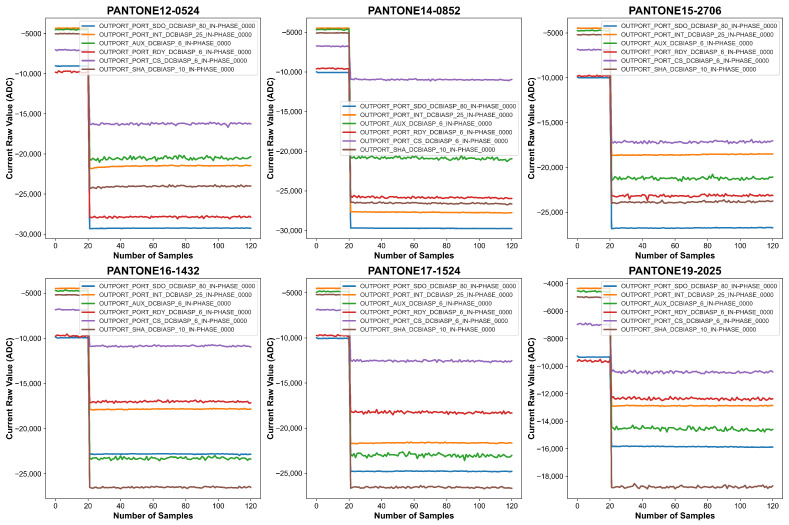
Graphical representation of the data collected for six PANTONE color cards with LCCW.

In particular, the active LED is excited by modulating its bias current with a sinusoidal waveform. The IN-PHASE component of the photodiode current is precisely detected, ensuring that artifacts from light sources other than the active LED are rejected. Each measurement corresponds to a specific LED configuration, with different wavelengths being stimulated to enhance the spectral differentiation of the PANTONE colors. The detailed LED configurations and corresponding wavelengths used in data acquisition are outlined in Table 2. This carefully controlled data collection process ensures that the dataset is well-suited for the application of machine learning algorithms in PANTONE color classification, laying the foundation for accurate and scalable color identification using wearable sensor technology. Figure 9 and Figure 10 provide visual representations of the spectral data collected under FCCW and LCCW, respectively. Under the firm contact condition, signal traces for each of the six channels are smooth, stable, and well-separated, reflecting the benefits of consistent sensor-surface proximity. In contrast, the loose contact condition introduces higher variability, likely caused by inconsistent pressure and air gaps; while the fundamental spectral characteristics are still present, signal fluctuations and overlap between channels are more prominent under LCCW. These figures clearly demonstrate the influence of mechanical contact stability on spectral signal quality and underscore the importance of a robust experimental setup. A summary of the full-scale data acquisition parameters is provided in Table 3.

### 3.3. Data Preprocessing

To ensure the quality, consistency, and suitability of the raw spectral data for machine learning classification, a structured preprocessing pipeline was implemented. The SENSIPATCH spectrometer module provides six raw input features per sample, corresponding to the in-phase current responses of the photodiode to sequential LED activations—four in the visible range and two in the near-infrared (NIR), as detailed earlier. These raw signals, while informative, are susceptible to baseline drift, environmental fluctuations, and amplitude variations across spectral channels. Therefore, preprocessing was essential to correct for sensor offsets, standardize the data scale, and enhance robustness for subsequent model training. The specific preprocessing steps applied baseline correction, bootstrapping for dataset expansion, and Z-score normalization, are described in the following subsections.

#### 3.3.1. Baseline Correction

Before processing the measurements, baseline correction was applied to remove the sensor’s inherent response when no external material was present. The first 21 measurements, recorded with no PANTONE color card placed on the sensor, were used to establish the natural baseline response of the sensor. These readings captured any systematic variations due to environmental conditions and sensor behavior in the absence of external stimuli. As shown in Figure 9 and Figure 10, there is a sudden spike in the measurements when we put the PANTONE color card on the sensor. To correct for this baseline effect, the mean of the first 21 readings was computed, and this value was subtracted from each of the subsequent 100 spectral measurements collected with the PANTONE color card in place.

Mathematically, baseline correction was applied as:

(1) $$X_{\mathrm{corrected},i}=X_{\mathrm{raw},i}-X_{\mathrm{baseline}\_\mathrm{mean}},\;\forall i\in \left\{22,23,\ldots ,121\right\}$$

where:

- $X_{\mathrm{raw},i}$ is the original sensor measurement for the *i*th data point,
- $X_{\mathrm{baseline}\_\mathrm{mean}}$ is the mean of the first 21 baseline measurements (when no card was present).
- $X_{\mathrm{corrected},i}$ is the baseline-corrected spectral response for the *i*th data point.

This step ensured that all color-specific spectral data were measured relative to a common baseline, improving reliability and consistency across all measurements.

**Figure 11:**
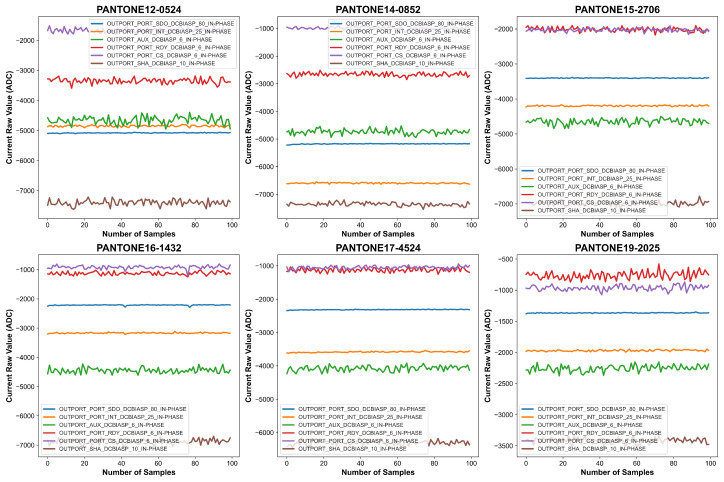
Graphical representation of the baseline-corrected data with firm contact condition for six PANTONE color cards.

**Figure 12:**
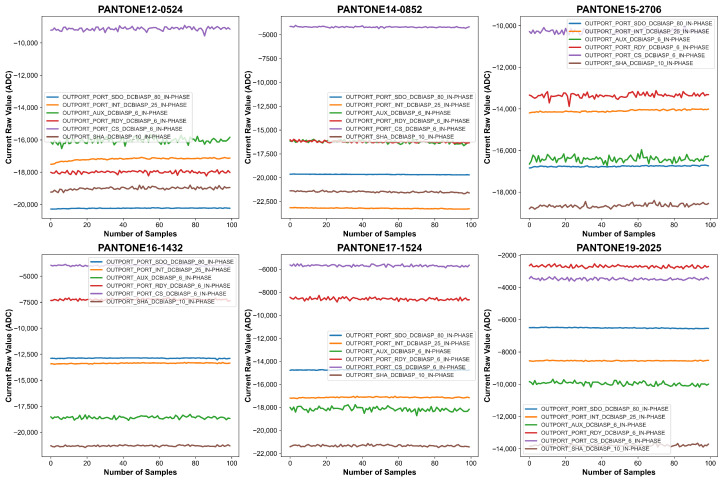
Graphical representation of the baseline-corrected data with loose contact condition for six PANTONE color cards.

The effects of this baseline correction procedure are graphically illustrated in Figure 11 and Figure 12, which shows baseline-adjusted signal curves for several representative color samples. The transformation from raw to corrected signals demonstrates enhanced alignment and reduced inter-channel variability. Notably, baseline-corrected data reveals clearer separation among channels and improved spectral stability, which is crucial for downstream machine learning tasks.

#### 3.3.2. Bootstrapping for Dataset Expansion

To enhance dataset robustness and mitigate overfitting, bootstrapping was employed as a data resampling technique. Each file, containing 100 data points after baseline correction, was resampled to generate 2000 data points. Bootstrapping was performed by randomly drawing spectral readings with replacement from the corrected dataset, ensuring a diverse and expanded dataset for analysis.

This resampling process maintained the same statistical distribution as the original data while ensuring sufficient variability for training the machine learning models. By generating an expanded dataset, the system became less sensitive to minor variations in spectral readings caused by sensor noise, ambient fluctuations, or experimental conditions. The increased dataset size also improved the model’s generalization ability and robustness against overfitting. Bootstrapping was applied only to the training set within each cross-validation fold, after the measurement-level train/test split, while the test set was kept unchanged. Its purpose was not to generate new physical information, but to provide a more robust resampled training distribution from the limited number of post-baseline-correction samples available for each measurement.

#### 3.3.3. Normalization Using Z-Score Standardization

After bootstrapping, Z-score normalization was applied to standardize spectral readings across different wavelengths and sensors. This transformation ensured that all spectral features had a mean of zero and unit variance, allowing for consistent feature scaling before applying machine learning models.

The Z-score normalization was computed using:

(2) $$X_{\mathrm{normalized}}=\frac{X_{\mathrm{bootstrapped}}-\mu }{\sigma }$$

where:

- $X_{\mathrm{bootstrapped}}$ is the spectral value after bootstrapping and baseline correction;
- μ is the mean of the resampled dataset;
- σ is the standard deviation of the resampled dataset.

For each cross-validation fold, the parameters required for Z-score normalization, namely the mean and standard deviation, were computed exclusively from the training data and then applied unchanged to the corresponding test data, thereby preventing data leakage. Since the six LED photodiode sensors operated at different wavelengths, this normalization helped mitigate variations in intensity levels across the different spectral bands, enabling the model to focus on spectral shape variations rather than absolute intensity differences.

### 3.4. Feature Importance Analysis of Multispectral Channels

In addition to normalization, we analyzed the contribution of each spectral channel using the built-in feature importance scores of the Random Forest classifier. Feature importance was computed for each cross-validation fold and averaged to obtain a stable ranking of the six wavelengths.

As shown in Table 4, the visible channels (468–645 nm) contribute most strongly to classification performance, with the red (645 nm) and yellow (593 nm) wavelengths exhibiting the highest importance. This is consistent with the physical basis of color discrimination, as the PANTONE classes are defined primarily in the visible spectrum. The near-infrared channels (850 nm and 950 nm) show lower but non-negligible importance, indicating that they provide complementary information related to material- and pigment-dependent reflectance properties.

### 3.5. Color Classification with Machine Learning

After data preprocessing, the next step in our methodology is the application of machine learning algorithms for the classification of PANTONE colors based on the spectral data acquired using the SENSIPATCH wearable system. The goal is to develop a robust model that accurately distinguishes between different colors while ensuring generalization across varying experimental conditions. As described in the previous subsection, each input sample to the machine learning models consists of a six-dimensional feature vector. The input features are used directly for training after the preprocessing. The classification task is defined as a supervised multi-class problem with 102 output classes. These include 100 standardized PANTONE color cards, one plain white paper sample, and one baseline class representing measurements without any object on the sensor.

#### Selection of Machine Learning Models

To ensure optimal classification of spectral responses corresponding to different PANTONE colors, we employed a range of machine learning models, each chosen based on its suitability for handling high-dimensional spectral data and its classification efficiency:

- Random Forest (RF): Selected for its robustness in handling high-dimensional sensor data and its interpretability via feature importance analysis. Its ensemble structure mitigates overfitting and enhances generalization capabilities [55,56].
- Support Vector Machine (SVM): Chosen due to its ability to construct complex decision boundaries, which is particularly advantageous for sensor-derived datasets where non-linear relationships may exist among features [57,58].
- Multilayer Perceptron (MLP): Selected as a classical neural network architecture capable of capturing non-linear relationships in numerical sensor data. Its fully connected structure enables the extraction of discriminative features from multivariate time series input, making it suitable for classification tasks involving wearable sensor measurements [59].
- Convolutional Neural Network (CNN): Employed due to its effectiveness in pattern recognition, especially when localized temporal or spatial features are relevant; while originally developed for image analysis, CNNs have shown strong performance on one-dimensional time-series data, such as those generated by sequential sensor readings, by leveraging convolutional filters to extract local structures [60].
- Long Short-Term Memory (LSTM): Utilized for its strength in modeling sequential dependencies, which is particularly beneficial when analyzing time-series sensor data where the order of readings conveys important contextual information. LSTM networks, a type of recurrent neural network (RNN), are designed to capture long-range dependencies and overcome vanishing gradient issues, making them suitable for tasks requiring temporal context modeling [61].

Among the evaluated models, the MLP demonstrated the most consistent generalization performance across both experimental conditions. Its flexibility in tuning and suitability for deployment in embedded systems further support its selection as the primary model in this study, while the Support Vector Machine (SVM) achieved the highest accuracy under firm contact conditions, and Random Forest performed competitively on the combined dataset, these models exhibited more limited adaptability across conditions. Table 5 summarizes the architectural configurations and training hyperparameters used for all models considered. These parameters were selected through empirical experimentation, where initial values were chosen based on standard practice and prior work in similar domains. To improve transparency, hyperparameter tuning was conducted iteratively using validation-based performance feedback, with the goal of maximizing classification accuracy while maintaining generalization across contact conditions. Specifically, practical ranges of learning rate, number of neurons, regularization strength, and training epochs were explored. Final configurations were selected based on convergence stability and consistent validation performance across folds; while no exhaustive hyperparameter search was conducted, the selected settings provided a reliable balance between model accuracy, computational efficiency, and robustness across varying contact scenarios.

## 4. Machine Learning Experiments and Results

**Figure 13:**
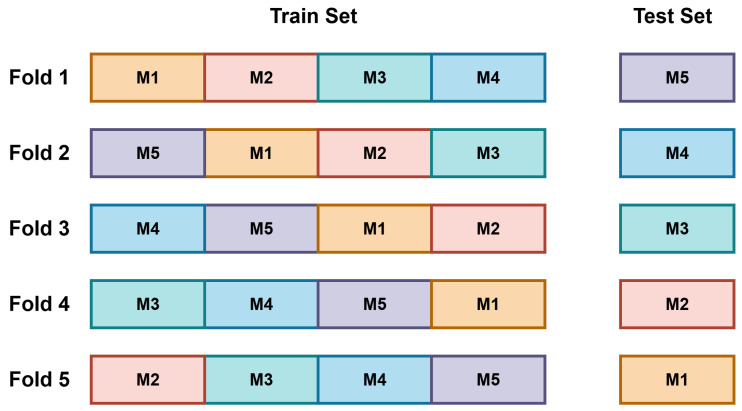
Stratified 5-fold cross-validation across 102 classes adopted for LCCW and FCCW data. Each row represents the fold, and each column represents the measurement. In each fold, one measurement per class is used for testing, and the remaining four are used for training.

To assess the performance of the proposed machine learning models, we conducted classification experiments under three experimental scenarios: FCCW, LCCW, and a combined condition (FCCW + LCCW). These scenarios reflect both ideal and real-world sensor placement configurations and allow evaluation of model robustness against variations in contact pressure. To ensure robust and unbiased performance evaluation, we adopted 5-fold cross-validation for both the FCCW and LCCW scenarios, as shown in Figure 13, and 10-fold cross-validation for the Combined condition. These fold counts were directly aligned with the number of available measurements per class: five measurements in FCCW and LCCW individually, and ten in the Combined condition (i.e., five from each contact type). In each fold, one measurement per class was held out for testing while the remaining were used for training, ensuring measurement-level independence and preventing data leakage. This fold-wise approach helped mitigate overfitting and allowed each model to be evaluated on all measurement sequences in a rotation-based manner.

For each of the 102 classes (comprising 100 PANTONE color cards, one white paper reference, and one baseline), five independent measurements were recorded under each contact condition. Each measurement initially consisted of 121 sensor readings, of which 100 were retained after baseline correction. To increase dataset size and improve model robustness, bootstrapping was applied to each corrected measurement, resulting in 2000 samples per measurement. This produced a total of 1,020,000 samples per condition (102 classes × 5 measurements × 2000 samples). In both FCCW and LCCW experiments, four out of the five measurements per class were used for training, and the remaining one was used for testing. Therefore, each condition included 8000 training samples per class (4 measurements × 2000 samples) and 2000 test samples per class (1 measurement × 2000 samples), leading to a total of 816,000 training samples and 204,000 test samples per condition.

**Figure 14:**
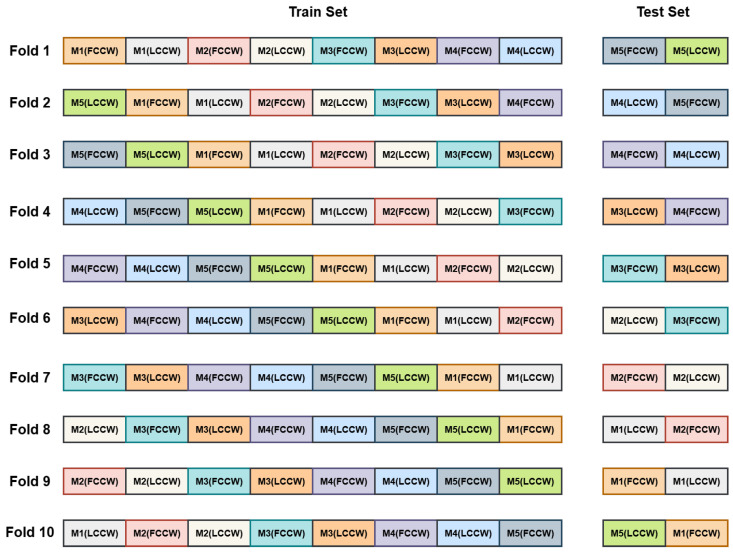
Stratified 10-fold cross-validation across 102 classes adopted for combined data. Each row represents the fold, and each column represents the measurement. In each fold, two measurements per class are used for testing from FCCW and LCCW, and the remaining eight are used for training.

In the Combined condition, all ten measurements per class (five from FCCW and five from LCCW) were utilized as shown in Figure 14. The training set comprised eight measurements per class, four from each contact condition, while the test set included the remaining two measurements (one from each condition). This resulted in 1,632,000 training samples (102 × 8 × 2000) and 408,000 test samples (102 × 2 × 2000). By incorporating both contact configurations in the training and evaluation phases, this setup enabled a more comprehensive and realistic assessment of model generalization under variable physical deployment scenarios.Table 6 summarizes the measurement structure, number of samples, and the corresponding cross-validation folds used for each experimental condition.

### 4.1. Classification Performance Under FCCW

This subsection evaluates model performance under the Firm Contact Condition With Weight (FCCW), representing a stable and ideal sensor–card interface. In this setup, a circular weight with a hollow center was used to apply uniform pressure on the PANTONE color card, minimizing air gaps and enhancing signal stability.

Among all models, SVM achieved the highest average accuracy, 97.29%, with Random Forest 97.17% and MLP 97.04% following closely. CNN also performed competitively, whereas LSTM showed significantly lower accuracy, indicating limited effectiveness in this static setup. Table 7 summarizes the five-fold cross-validation results.

To further analyze model behavior, Table 8 presents the mean precision, recall, and F1 score for each classifier.

### 4.2. Classification Performance Under LCCW

This subsection evaluates model performance under the Loose Contact Condition Without Weight (LCCW), which simulates real-world wearable usage scenarios involving potential card misalignment and movement. In this setup, no weight was applied to the PANTONE color card, leading to reduced contact stability and increased signal variability.

Despite these challenges, most models maintained high classification accuracy, as shown in Table 9. SVM achieved the highest mean accuracy of 97.14%, outperforming all other models under LCCW. MLP followed with a mean accuracy of 95.86%, while CNN and RF recorded 93.26% and 91.84%, respectively. LSTM again showed the weakest performance, with a mean accuracy of 76.33%, indicating limited robustness to contact variation.

To further assess model performance, Table 10 reports the average precision, recall, and F1 scores. SVM continued to lead with an F1 score of 96.83%, reflecting strong generalization in the presence of contact inconsistency. MLP also performed well with an F1 score of 95.05%, reinforcing its robustness. CNN and RF followed, while LSTM remained the least effective model across all metrics.

These results suggest that while reduced contact stability does introduce classification variability, well-regularized models like SVM and MLP can maintain high performance. This reinforces the need for models that can tolerate minor sensor–card misalignment in real-world deployments.

### 4.3. Performance Under Combined Conditions (FCCW + LCCW)

To evaluate model robustness under realistic and variable sensor contact conditions, datasets from both FCCW and LCCW scenarios were merged to form a comprehensive dataset. This setup simulates practical deployment scenarios where contact pressure and alignment may vary. Classification performance was evaluated using 10-fold cross-validation, with results summarized in Table 11.

Among the models, the MLP achieved the highest mean accuracy 96.08%, followed by Random Forest 94.96%. In contrast, SVM performance dropped notably to 88.40%, highlighting its sensitivity to contact variation. CNN and LSTM recorded lower average accuracies of 84.18% and 70.21%, respectively, with LSTM once again demonstrating the weakest performance across folds.

Table 12 presents the average precision, recall, and F1 score for each model under the combined condition. MLP maintained its leading position with the highest F1 score 95.37%, indicating strong generalization to varying contact dynamics. RF also performed well with an F1 score of 93.85%. Meanwhile, SVM’s decline in recall suggests reduced robustness under mixed-contact conditions. CNN showed moderate performance, and LSTM remained consistently the least effective.

These results confirm that training on combined datasets enables better generalization across heterogeneous contact conditions. MLP’s superior performance highlights its adaptability and makes it a strong candidate for deployment in real-world, variable-pressure environments. The drop in SVM’s performance further supports the need for models that can accommodate signal inconsistencies introduced by imperfect sensor placement.

### 4.4. Discussion and Comparative Insights

**Figure 15:**
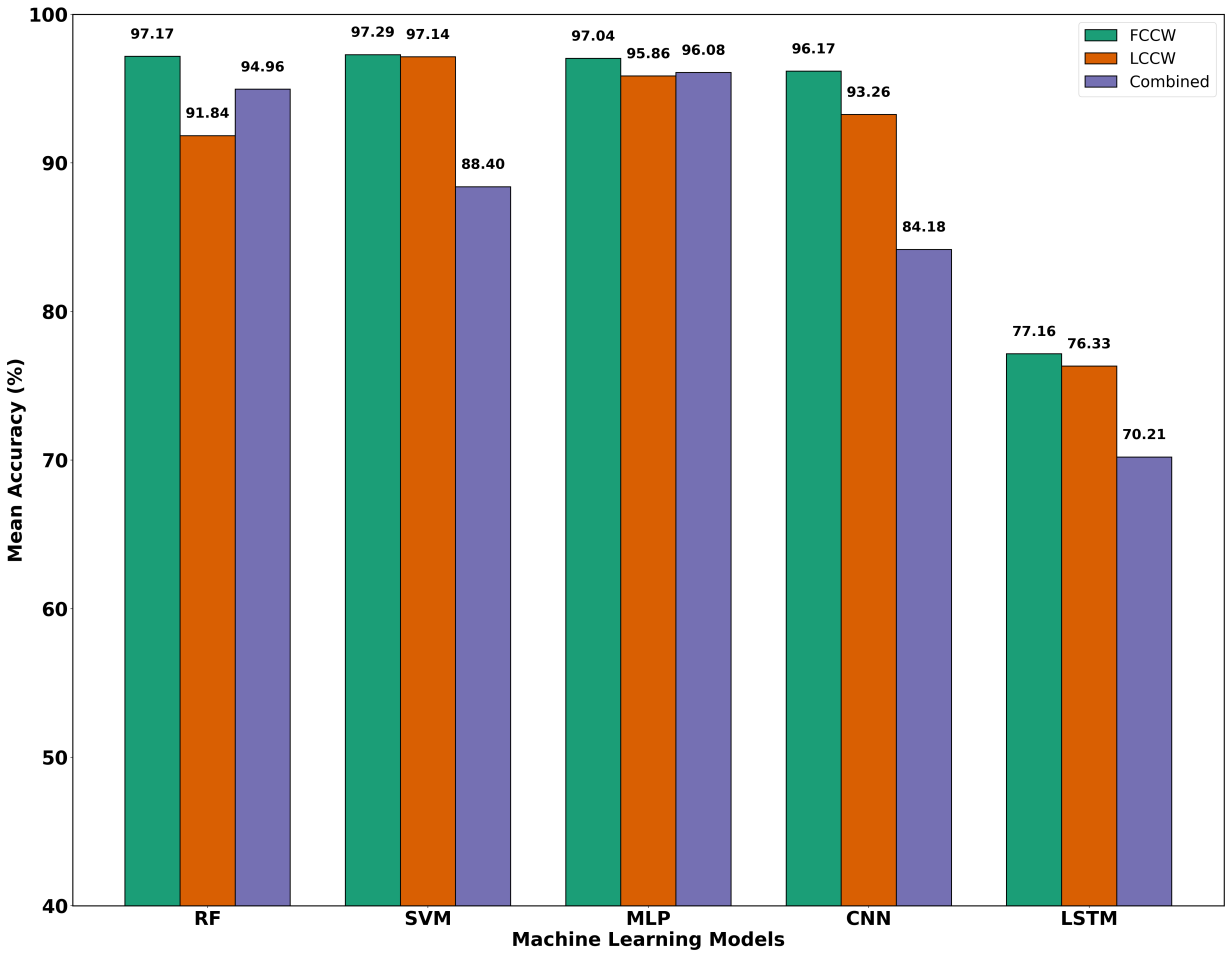
Classification performance of machine learning models under Firm Contact Condition With Weight (FCCW), Loose Contact Condition Without Weight (LCCW), and combined conditions.

The experimental results reveal that machine learning models can effectively classify spectral responses from the SENSIPATCH wearable system across varying contact scenarios. Figure 15 presents the performance of the machine learning models under FCCW, LCCW, and combined contact conditions. Among all models, the MLP consistently demonstrated strong and stable performance across all evaluation settings, particularly excelling in the combined setup with a mean accuracy of 96.08%. SVM also achieved competitive results, slightly outperforming MLP under the LCCW, while RF delivered strong baseline performance, particularly in stable environments. CNN exhibited reasonable generalization and handled noisy data better than LSTM, although its overall performance was marginally lower than that of MLP and SVM. LSTM networks, while suited to sequential data modeling, proved less effective in this context. This may be due to the relatively short sequence length and the absence of strong temporal dependencies in the spectral signal, which limits the advantage of recurrent architectures. Contact pressure was found to play a significant role in classification stability. The FCCW produced more consistent and well-separated spectral signatures, reflected in higher model performance across the board. However, the performance drop under LCCW was relatively modest, indicating that the system maintains resilience to moderate physical misalignment. This reinforces the importance of designing sensor systems and corresponding algorithms that tolerate real-world variability in deployment.

Furthermore, fold-to-fold variability observed in the LCCW and combined datasets underscores the value of training on diverse conditions. By incorporating both contact scenarios into a single dataset, models were better able to generalize across different sensor placements, which is critical for wearable applications. This combined training approach simulates realistic deployment conditions and prepares the model for variations in skin contact pressure and user motion. Among the evaluated models, the MLP was selected as the primary classifier for deployment due to its strong balance between classification accuracy, generalization capability, and architectural flexibility, while SVM demonstrated slightly higher accuracy under stable contact conditions, and RF performed competitively on the combined dataset, MLP consistently exhibited robust performance across both firm and loose contact scenarios. Moreover, MLPs are well-suited for iterative fine-tuning, scalable learning with larger datasets, and efficient integration into embedded systems through techniques such as pruning and quantization. This behavior can be attributed to the nature of the distortions introduced by contact variability in the sensing process. Changes in contact pressure do not result in uniform scaling of the signal but instead produce uneven variations across the six spectral channels, altering their relative relationships. In this context, the MLP is able to exploit these inter-channel patterns by learning combinations of spectral responses rather than relying on individual feature magnitudes. In contrast, models such as SVM operate on a fixed representation of the input space and are more sensitive to such non-uniform shifts, particularly when the relative structure of the features changes between training and testing conditions. This makes the MLP more suitable for handling the type of variability introduced by loose-contact measurements in the proposed system. These practical advantages, combined with their adaptability to real-world sensor variability, support the choice of MLP as the preferred model in this study.

**Figure 16:**
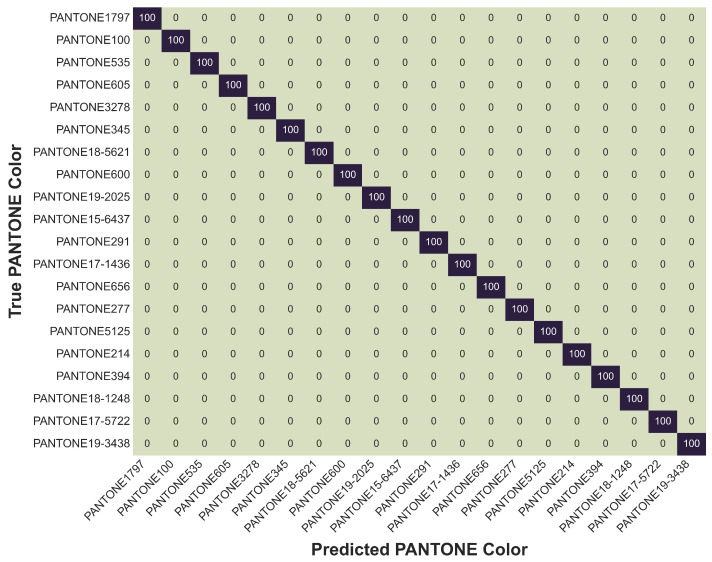
Subset confusion matrix of the MLP model trained on combined condition data, illustrating selected PANTONE color classes with high classification accuracies.

**Figure 17:**
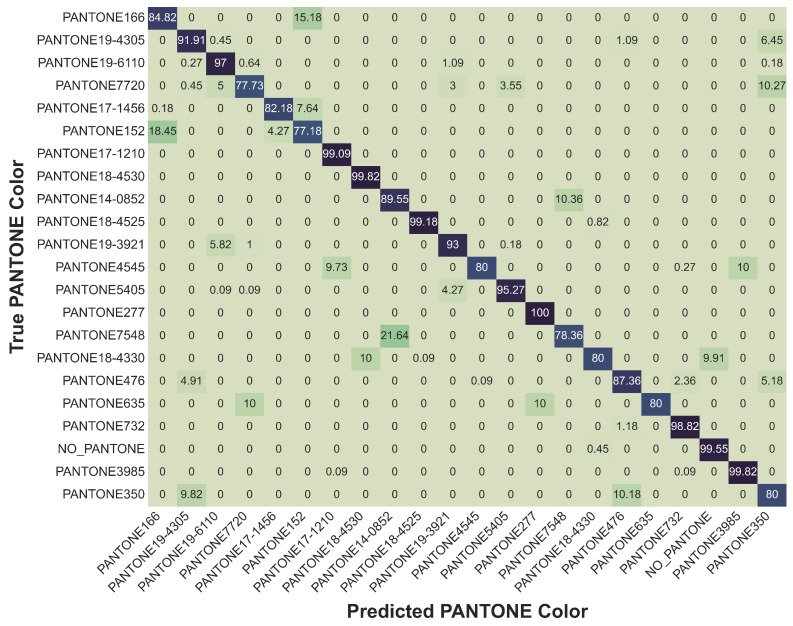
Subset confusion matrix for the MLP model trained on combined condition data, focusing on misclassification among PANTONE color classes with the lowest accuracy.

To gain a deeper understanding of the classification behavior at the class level, we analyzed the model’s performance using both subset confusion matrices and a detailed class-wise accuracy table. Given the large number of classes (102), presenting the full confusion matrix was impractical. Instead, two targeted confusion matrices were generated: one highlighting classes with the highest accuracies (Figure 16) and another focusing on classes with the lowest accuracies (Figure 17).

**Figure 18:**
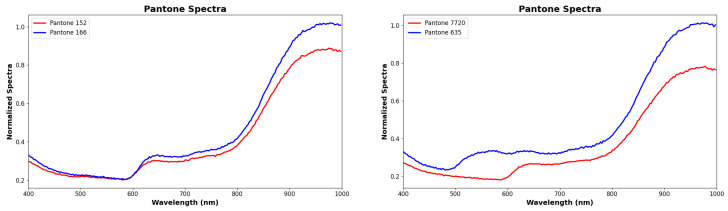
Normalized spectra of selected challenging PANTONE pairs. **Left**: PANTONE152 and PANTONE166. **Right**: PANTONE7720 and PANTONE635. In both cases, the spectra show limited separability across the measured wavelength range, helping to explain the observed misclassifications.

The confusion matrix for the lower-performing classes revealed that certain colors, such as PANTONE166, PANTONE7720, and PANTONE17-1456, exhibited greater misclassification rates. These misclassifications were predominantly observed between spectrally similar tones, notably between PANTONE166 and PANTONE152, or between PANTONE7720 and PANTONE635. Such overlaps are not unexpected, given the subtle differences in their reflectance spectra, particularly within muted red and dark green regions. Interestingly, some confusion was also noted between dark-toned samples and the NO_PANTONE baseline class, suggesting that very low reflectance levels can sometimes blur the model’s decision boundaries. Nevertheless, even among these more challenging classes, the MLP model maintained respectable classification performance, with accuracies consistently above 75%. To further clarify this behavior, Figure 18 shows representative spectral comparisons for selected challenging PANTONE pairs. In particular, the spectra of PANTONE152 and PANTONE166 exhibit very similar trends across the measured wavelength range, with only limited separation. Because the current SENSIPATCH spectrometer module relies on a small number of discrete optical channels, such subtle spectral differences are not always captured with sufficient resolution at the sensor level. Consequently, these classes may generate overlapping multispectral responses, which reduces the discriminative information available to the classifier and increases the likelihood of misclassification. The same figure also shows the spectral comparison between PANTONE7720 and PANTONE635, indicating that the observed difficulty is mainly associated with high spectral similarity between specific color pairs, while low overall reflectance may further reduce separability in some darker tones.

In contrast, the subset confusion matrix of high-performing classes showcased exemplary classification results. Twenty representative colors, including vivid tones such as PANTONE1797, PANTONE345, and PANTONE535 were classified with 100% accuracy, without any observable misclassifications. These results demonstrate the model’s strong ability to distinguish between spectrally distinct colors, a feature that holds substantial promise for real-world wearable applications. It is worth noting that although only 20 classes are shown in the figure for clarity, a much larger number of classes actually achieved perfect classification. To complement the confusion matrices, Table 13 provides a comprehensive view of the class-wise classification accuracies for all PANTONE color classes. The table reveals that an overwhelming majority of classes achieved exceptionally high accuracy, with 65 classes classified at 99% or above. Highly saturated and spectrally well-separated colors, such as PANTONE100, PANTONE1797, PANTONE3278, and WHITE_PAPER, were reliably distinguished by the MLP model. Conversely, a smaller group of colors particularly PANTONE7720, PANTONE166, PANTONE152, and PANTONE635 achieved lower, though still reasonable, accuracies between 77% and 85%. These findings align well with observations from the confusion matrix analysis, where muted or spectrally overlapping colors posed greater challenges.

Taken together, the insights from the subset confusion matrices and the class-wise accuracy table illustrate a consistent trend: when colors exhibit distinct and strong spectral features, classification is highly reliable. In cases where spectral differences are more nuanced, performance declines modestly but remains robust. Overall, the SENSIPATCH system, combined with a well-designed machine learning pipeline, demonstrates excellent potential for scalable, reliable, and real-world colorimetric sensing applications.

## 5. Conclusions and Future Works

This study presented a machine-learning-driven framework for accurate color classification using the SENSIPATCH, a compact, wearable spectral sensing system based on multi-wavelength LED–photodiode configurations. Through a structured experimental protocol involving firm and loose contact scenarios, we demonstrated the SENSIPATCH’s capability to capture stable and distinctive spectral signatures across 100 PANTONE color classes. Among the five machine learning models evaluated, the multilayer perceptron (MLP) consistently delivered the strongest generalization performance across both contact conditions, achieving a mean accuracy of 96.08% under combined scenarios. These results underscore the potential of integrating compact spectroscopic sensing with machine learning for practical, real-time color recognition in wearable applications. Graphical analyses of baseline-corrected and raw spectral data confirmed the importance of contact pressure and validated the effectiveness of the preprocessing pipeline. However, several limitations remain. The current system was evaluated in a controlled, enclosed environment designed to minimize ambient interference and ensure repeatability; while effective for benchmarking, this setting does not fully replicate real-world deployment conditions, where ambient lighting, motion artifacts, and sensor alignment variability can influence spectral readings. Preliminary evaluations from our previous work [16] indicated stable performance under ambient lighting conditions, but further studies are needed to assess robustness under dynamic, uncontrolled environments. As part of future work, we plan to conduct additional experiments in open settings to evaluate performance across varying illumination, motion, and environmental conditions, as well as to investigate device-to-device consistency among multiple SENSIPATCH units. Another important consideration is surface geometry and compliance. The current study utilized flat, matte PANTONE color cards to ensure standardized and repeatable measurements. However, many real-world materials, such as skin, textiles, plastics, and food items, exhibit curvature, texture, gloss, or mechanical compliance. The system’s use of multiple directional LEDs and normalization techniques inherently provides some tolerance to such geometric and structural variations. Nonetheless, the current study is limited to flat, matte samples, and future work will include dedicated experiments on non-planar, textured, and compliant surfaces to extend the system’s applicability to more realistic materials and practical use cases.

Model-wise, while the MLP performed effectively in this study, further optimization is necessary to ensure compatibility with ultra-low-power microcontroller platforms suitable for embedded wearable applications. We also acknowledge that the current hyperparameter tuning relied on empirical experimentation. Future iterations will incorporate systematic optimization strategies, such as grid search, random search, or Bayesian optimization, to improve reproducibility and performance. Additionally, enhancing model interpretability will be an important step, particularly for applications in biomedical or safety-critical domains. Finally, Table 14 compares the main technical characteristics of our system with the latest generation of commercial devices. It should be noted that SENSIPATCH is larger than the other products used for comparison, but this is due to the fact that some biomedical applications require large areas of investigation. Although scanning times are comparable, the present prototype is lighter, less expensive, and multi-purpose. Finally, thanks to the LEDs chosen, the range of wavelengths covered is wider.

## Figures and Tables

**Table 1:** List of PANTONE Color Codes Used in the Study. * NO_PANTONE corresponds to baseline sensor measurements without a card. WHITE_PAPER refers to plain white paper used as a reference sample.

No.	PANTONE Code	No.	PANTONE Code	No.	PANTONE Code	No.	PANTONE Code
1	NO_PANTONE *	2	PANTONE100	3	PANTONE108	4	PANTONE11-4804
5	PANTONE12-0313	6	PANTONE12-0524	7	PANTONE12-0727	8	PANTONE12-5204
9	PANTONE13-0645	10	PANTONE13-0850	11	PANTONE13-1107	12	PANTONE13-5304
13	PANTONE14-0848	14	PANTONE14-0852	15	PANTONE14-1119	16	PANTONE14-1419
17	PANTONE14-1513	18	PANTONE14-1911	19	PANTONE14-3612	20	PANTONE14-4516
21	PANTONE142	22	PANTONE15-0543	23	PANTONE15-0942	24	PANTONE15-1247
25	PANTONE15-1816	26	PANTONE15-2706	27	PANTONE15-5210	28	PANTONE15-6437
29	PANTONE152	30	PANTONE16-1255	31	PANTONE16-1329	32	PANTONE16-1432
33	PANTONE16-1439	34	PANTONE166	35	PANTONE17-1210	36	PANTONE17-1436
37	PANTONE17-1456	38	PANTONE17-1564	39	PANTONE17-4021	40	PANTONE17-4524
41	PANTONE17-5722	42	PANTONE1797	43	PANTONE18-0119	44	PANTONE18-1248
45	PANTONE18-2326	46	PANTONE18-3025	47	PANTONE18-4330	48	PANTONE18-4525
49	PANTONE18-4530	50	PANTONE18-5621	51	PANTONE19-1250	52	PANTONE19-2025
53	PANTONE19-3438	54	PANTONE19-3921	55	PANTONE19-3935	56	PANTONE19-4305
57	PANTONE19-6110	58	PANTONE199	59	PANTONE214	60	PANTONE277
61	PANTONE291	62	PANTONE3278	63	PANTONE345	64	PANTONE350
65	PANTONE383	66	PANTONE394	67	PANTONE3985	68	PANTONE414
69	PANTONE4545	70	PANTONE466	71	PANTONE4685	72	PANTONE476
73	PANTONE485	74	PANTONE493	75	PANTONE5125	76	PANTONE535
77	PANTONE5405	78	PANTONE5555	79	PANTONE583	80	PANTONE600
81	PANTONE605	82	PANTONE635	83	PANTONE656	84	PANTONE677
85	PANTONE691	86	PANTONE732	87	PANTONE7415	88	PANTONE7493
89	PANTONE7548	90	PANTONE7590	91	PANTONE7654	92	PANTONE7720
93	PANTONE7726	94	PANTONE7737	95	PANTONE9041	96	PANTONE9044
97	PANTONE9180	98	PANTONE9241	99	PANTONE9320	100	PANTONECoolGray7
101	PANTONEWarmGray2	102	WHITE_PAPER *				

**Table 2:** LED features and measurement configurations. The columns represent feature name, LED color (LC), and wavelength (WL) in nanometers.

Feature Name	LC	WL
OUTPORT PORT SDO DCBIASP 80 IN-PHASE	Yellow	593
OUTPORT PORT INT DCBIASP 25 IN-PHASE	Red	645
OUTPORT AUX DCBIASP 6 IN-PHASE	Infrared	950
OUTPORT SHA DCBIASP 10 IN-PHASE	Infrared	850
OUTPORT PORT RDY DCBIASP 6 IN-PHASE	Green	523
OUTPORT PORT CS DCBIASP 6 IN-PHASE	Blue	468

**Table 3:** Summary of data collection for 100 PANTONE color dataset.

Parameter	Value/Description
Number of PANTONE Colors	100
Additional Sample	Plain White Paper
Trials per Color	5
Sensor Readings per Trial	121
Baseline Samples per Trial	21
Experimental Conditions	2 (Firm Contact—FCCW, Loose Contact—LCCW)
Total Samples	123,420 (102 × 5 × 121 × 2)
Photodiode Read Time per Light Source	265 ms
Light Sources Used	6 (4 LEDs + 2 NIR)
Time per Full Acquisition Cycle	1.6 s
Sampling Rate	0.62 full cycles/s
Sensor Output Components	In-Phase (used), Quadrature (available)
LED Configuration	See Table 2
Contact Conditions	FCCW (firm), LCCW (loose)

**Table 4:** Random Forest feature importance of the six multispectral channels under the combined condition. Values are reported as mean ± standard deviation across cross-validation folds.

Channel	Wavelength	Mean Importance	Std
Red	645 nm	0.2699	0.0035
Yellow	593 nm	0.2471	0.0064
Blue	468 nm	0.1605	0.0022
NIR	850 nm	0.1332	0.0021
Green	523 nm	0.1213	0.0021
NIR	950 nm	0.0680	0.0029

**Table 5:** Architectural configurations and training hyperparameters of models used.

Parameter	MLP	Random Forest	SVM	2D CNN	LSTM
Key Layers	Dense (100, 50)	–	–	Conv2D (16, 32), Dense (64)	LSTM (32, 32), Dense (32)
Activation Function	ReLU	–	–	ReLU	ReLU (Dense)
Optimizer	Adam	–	–	Adam	Adam
Learning Rate	–	–	–	0.001	0.001
Regularization	α=0.001	–	–	–	–
Loss Function	Categorical Cross-Entropy	–	–	Sparse Categorical Cross-Entropy	Sparse Categorical Cross-Entropy
Batch Size	64	–	–	32	16
Epochs	50	–	–	20	20
Window Size	–	–	–	5	5
Additional Notes	MaxIter = 500	200 Estimators	Linear Kernel, Prob = True	Dropout (0.5), BatchNorm	Dropout (0.5), BatchNorm

**Table 6:** Bootstrapped dataset structure, sample distribution, and cross-validation setup.

Attribute	FCCW	LCCW	Combined
Measurements per Class	5	5	10 (5 + 5)
Samples per Measurement (after bootstrapping)	2000	2000	2000
Training Samples	816,000 (102 × 4 × 2000)	816,000 (102 × 4 × 2000)	1,632,000 (102 × 8 × 2000)
Test Samples	204,000 (102 × 1 × 2000)	204,000 (102 × 1 × 2000)	408,000 (102 × 2 × 2000)
Cross-Validation Folds	5	5	10

**Table 7:** Classification accuracy under FCCW (%).

Model	Fold-1	Fold-2	Fold-3	Fold-4	Fold-5	Mean
RF	98.09	96.99	98.70	96.34	95.75	97.17
SVM	96.58	97.62	98.64	96.12	97.49	97.29
MLP	96.85	96.53	98.61	96.17	97.05	97.04
LSTM	76.71	79.38	79.36	77.43	72.29	77.16
CNN	92.94	98.90	98.63	98.20	92.19	96.17

**Table 8:** Mean performance metrics for ML models under FCCW.

Model	Precision	Recall	F1 Score
RF	97.53	97.17	96.91
MLP	97.31	97.04	96.74
SVM	97.48	97.29	97.09
CNN	94.83	96.17	95.11
LSTM	68.87	77.16	71.21

**Table 9:** Classification accuracy under LCCW (%).

Model	Fold-1	Fold-2	Fold-3	Fold-4	Fold-5	Mean
RF	89.19	96.93	94.77	91.89	86.43	91.84
SVM	96.05	98.80	99.00	97.80	94.05	97.14
MLP	94.04	98.90	97.99	95.92	92.49	95.86
LSTM	74.73	79.29	78.60	77.77	71.27	76.33
CNN	91.77	92.01	94.61	92.81	95.13	93.26

**Table 10:** Mean performance metrics for ML models under LCCW.

Model	Precision	Recall	F1 Score
RF	90.48	91.80	90.10
MLP	95.58	95.84	95.05
SVM	97.41	97.13	96.83
CNN	93.12	94.83	93.39
LSTM	69.39	76.33	70.97

**Table 11:** Classification accuracy for combined conditions (%).

Fold	RF	SVM	MLP	LSTM	CNN
1	98.90	85.93	97.96	65.51	85.27
2	97.72	87.19	97.63	64.38	79.43
3	97.82	87.05	97.74	62.66	84.57
4	93.19	85.65	95.52	67.59	80.61
5	93.19	84.11	94.29	71.81	77.86
6	88.87	85.86	89.27	71.05	84.24
7	98.43	92.56	97.26	76.21	85.91
8	97.40	93.72	99.30	74.74	88.69
9	93.34	93.75	97.46	74.38	91.33
10	89.47	88.14	88.14	73.84	83.89
Mean	94.96	88.40	96.08	70.21	84.18

**Table 12:** Mean performance metrics for ML models for combined conditions (%).

Model	Precision	Recall	F1 Score
Random Forest	94.44	94.96	93.85
SVM	86.46	88.40	85.74
MLP	96.04	96.08	95.37
CNN	82.65	84.18	80.77
LSTM	61.77	70.21	62.81

**Table 13:** MLP model class-wise accuracy across PANTONE color classes for combined conditions.

Class	Accuracy (%)	Class	Accuracy (%)	Class	Accuracy (%)
NO_PANTONE	99.55	PANTONE100	100.0	PANTONE108	100.0
PANTONE11-4804	99.73	PANTONE12-0313	100.0	PANTONE12-0524	99.82
PANTONE12-0727	100.0	PANTONE12-5204	83.0	PANTONE13-0645	100.0
PANTONE13-0850	99.82	PANTONE13-1107	95.82	PANTONE13-5304	99.91
PANTONE14-0848	100.0	PANTONE14-0852	89.55	PANTONE14-1119	100.0
PANTONE14-1419	99.91	PANTONE14-1513	87.91	PANTONE14-1911	100.0
PANTONE14-3612	96.36	PANTONE14-4516	99.91	PANTONE142	99.82
PANTONE15-0543	100.0	PANTONE15-0942	90.0	PANTONE15-1247	100.0
PANTONE15-1816	99.09	PANTONE15-2706	99.91	PANTONE15-5210	89.91
PANTONE15-6437	100.0	PANTONE152	77.18	PANTONE16-1255	100.0
PANTONE16-1329	99.73	PANTONE16-1432	99.91	PANTONE16-1439	89.73
PANTONE166	84.82	PANTONE17-1210	99.09	PANTONE17-1436	100.0
PANTONE17-1456	82.18	PANTONE17-1564	97.36	PANTONE17-4021	99.82
PANTONE17-4524	99.91	PANTONE17-5722	100.0	PANTONE1797	100.0
PANTONE18-0119	86.18	PANTONE18-1248	100.0	PANTONE18-2326	99.91
PANTONE18-3025	97.91	PANTONE18-4330	80.0	PANTONE18-4525	99.18
PANTONE18-4530	99.82	PANTONE18-5621	100.0	PANTONE19-1250	98.36
PANTONE19-2025	100.0	PANTONE19-3438	100.0	PANTONE19-3921	93.0
PANTONE19-3935	98.36	PANTONE19-4305	91.91	PANTONE19-6110	97.0
PANTONE199	97.18	PANTONE214	100.0	PANTONE277	100.0
PANTONE291	100.0	PANTONE3278	100.0	PANTONE345	100.0
PANTONE350	80.0	PANTONE383	90.0	PANTONE394	100.0
PANTONE3985	99.82	PANTONE414	96.36	PANTONE4545	80.0
PANTONE466	89.91	PANTONE4685	91.36	PANTONE476	87.36
PANTONE485	98.64	PANTONE493	99.73	PANTONE5125	100.0
PANTONE535	100.0	PANTONE5405	95.27	PANTONE5555	96.73
PANTONE583	83.73	PANTONE600	100.0	PANTONE605	100.0
PANTONE635	80.0	PANTONE656	100.0	PANTONE677	99.82
PANTONE691	99.91	PANTONE732	98.82	PANTONE7415	99.18
PANTONE7493	100.0	PANTONE7548	78.36	PANTONE7590	100.0
PANTONE7654	91.36	PANTONE7720	77.73	PANTONE7726	100.0
PANTONE7737	100.0	PANTONE9041	100.0	PANTONE9044	99.0
PANTONE9180	99.91	PANTONE9241	90.36	PANTONE9320	99.82
PANTONECoolGray7	99.18	PANTONEWarmGray2	99.91	WHITE_PAPER	100.0

**Table 14:** Comparative overview of SENSIPATCH versus commercial portable colorimeters.

Device	Spectral Range	Acquisition Time	Size/Form Factor	Weight	Approx. Cost (EUR)	Additional Features
SENSIPATCH (this work)	468–950 nm (6 LED channels)	2 s per full cycle	18 × 18 cm patch	30 g	<50 (component-level)	Wearable, multi-sensor (bio-impedance, thermal, humidity)
highlightingNix Mini 3 [62]	400–700 nm	3 s per scan	Ø 40 mm × 40 mm	70 g	100–130	Bluetooth handheld, single-purpose
ColorMuse 3 [63]	400–700 nm	3.8 s per scan	Ø 43 mm × 45 mm	65 g	110–130	Bluetooth handheld, single-purpose

## Data Availability

Data will be available upon request.
